# miR-22 suppresses tumorigenesis and improves radiosensitivity of breast cancer cells by targeting Sirt1

**DOI:** 10.1186/s40659-017-0133-8

**Published:** 2017-09-07

**Authors:** Xia Zhang, Yuehua Li, Dan Wang, Xiaoer Wei

**Affiliations:** Department of Diagnostic and Interventional Radiology, Shanghai Jiao Tong University Affiliated Sixth People’s Hospital, No. 600 Yi Shan Road, Shanghai, 200233 China

**Keywords:** miR-22, Tumorigenesis, Radiosensitivity, Breast cancer, Sirt1

## Abstract

**Background:**

miR-22 has been shown to be frequently downregulated and act as a tumor suppressor in multiple cancers including breast cancers. However, the role of miR-22 in regulating the radioresistance of breast cancer cells, as well as its underlying mechanism is still not well understood.

**Methods:**

The expressions of miR-22 and sirt1 at mRNA and protein levels were examined by qRT-PCR and Western Blot. The effects of miR-22 overexpression and sirt1 knockdown on cell viability, apoptosis, radiosensitivity, γ-H2AX foci formation were evaluated by CCK-8 assay, flow cytometry, colony formation assay, and γ-H2AX foci formation assay, respectively. Luciferase reporter assay and qRT-PCR analysis were performed to confirm the interaction between miR-22 and sirt1.

**Results:**

miR-22 was downregulated and sirt1 was upregulated at both mRNA and protein levels in breast cancer cells. miR-22 overexpression or sirt1 knockdown significantly suppressed viability, induced apoptosis, reduced survival fraction, and increased the number of γ-H2AX foci in breast cancer cells. Sirt1 was identified as a target of miR-22 and miR-22 negatively regulated sirt1 expression. Ectopic expression of sirt1 dramatically reversed the inhibitory effect of miR-22 on cell viability and promotive effect on apoptotic rates and radiosensitivity in breast cancer cells.

**Conclusions:**

miR-22 suppresses tumorigenesis and improves radiosensitivity of breast cancer cells by targeting sirt1, providing a promising therapeutic target for breast cancer.

## Background

Breast cancer is the most commonly diagnosed malignancy globally, ranking second in cancer-related mortality in women [1]. Despite the advances in both diagnosis and comprehensive treatment of breast cancer, there are still 500,000 deaths of breast cancer per year worldwide [2]. It is estimated that approximately 25% of new cases will be diagnosed with breast cancer in 2015 [3]. It is well known that radiotherapy is currently a major adjuvant treatment for the majority of breast cancer patients [4]. This strategy helps to reduce the risk of recurrence by 70% and improve survival of breast cancer patients [5]. However, radioresistance is a major challenge to attaining maximal efficacy for successful radiotherapy of breast cancers [6]. Therefore, better understanding the underlying mechanisms involved in radioresistance and developing more effective therapeutic strategy are essential and urgent.

MicroRNAs (miRNAs) represent a group of small non-coding RNAs that negatively regulate the expression of multiple target genes at the post-transcriptional level, either through mRNA degradation or translational inhibition [7]. Aberrant expression of miRNAs has been demonstrated in various tumors including breast cancer [8]. These miRNAs, functioning as either oncogenes or tumor suppressors, are involved in tumorigenesis and progression of breast cancer [9]. Importantly, miRNA has previously been shown to play a critical role in modulating radioresistance of breast cancer cells [10]. For example, miR-668 overexpression enhanced radiosensitivity of breast cancer cells by targeting NF-κB inhibitor IκBα [11]. Ectopic expression of miR-129 sensitized breast cancer cells to irradiation and suppressed irradiation-induced autophagy [12]. Overexpression of miR-144 increased radioresistance of breast cancer cells by promoting proliferation, migration and invasion [10]. Recently, miR-22 has been shown to be frequently downregulated and act as a tumor suppressor in several cancers including breast cancers [13, 14]. However, the role of miR-22 in regulating the radioresistance of breast cancer cells, as well as its underlying mechanism is still unknown.

Silent information regulator 1 (Sirt1), a class III histone deacetylase, is the mammalian homolog of yeast Sirt2, which regulates chromatin silencing in yeast [15, 16]. Sirt1 has been emerged as a crucial regulator in many physiological processes, such as aging, differentiation, apoptosis, DNA damage and tumor development in mammalians [17, 18]. A previous document found that sirt1 was upregulated in breast cancer [19]. Moreover, it was reported that sirt1 deficiency suppressed the formation of repair foci which lead to DNA damage, thereby increasing the number of cancer cells undergoing apoptosis [20]. Sirt1 has been identified as a direct target of miR-22 in mouse ovarian granulosa cells [21], glioblastoma cells [22], as well as renal cell carcinoma [23]. However, whether miR-22 could directly target sirt1 in breast cancer is unclear.

In the present study, we aimed to investigate the roles of miR-22 and sirt1 in tumorigenesis and radioresistance of breast cancer cells. Furthermore, we confirm the interaction between miR-22 and sirt1 in breast cancer cells.

## Methods

### Cell lines and culture

Human breast cancer cell lines (MDA-MB-231 and MCF-7) and normal breast epithelial cell line MCF-10A were purchased from the American Type Culture Collection (ATCC, Manassas, VA, USA). MDA-MB-231 and MCF-7 cells were cultured in RPMI-1640 medium (Invitrogen, Carlsbad, CA, USA) containing 10% heat-inactivated fetal calf serum (FBS; Invitrogen), 100 U/mL penicillin, and 100 μg/mL streptomycin (Invitrogen). MCF-10A cells were grown in Ham’s F12: DMEM (1:1) medium (Gibco, Grand Island, NY, USA) containing 2 mM l-glutamine, 100 ng/mL epidermal growth factor (EGF) (Sigma, St. Louis, MO, USA), 0.1 mg/ml cholera toxin (CT; Sigma), 10 μg/ml insulin (Sigma), 500 ng/ml hydrocortisone (Sigma) and 5% horse serum (Atlanta Biologicals). All cells were cultured in 5% CO_2_ at 37 °C.

### Cell transfection

miR-22 mimics (miR-22), miR-22 inhibitor (anti-miR-22), scrambled negative control miRNA (miR-NC), siRNA specific targeting sirt1 (si-sirt1), scrambled negative control siRNA (si-NC), and plasmid encoding sirt1 (pcDNA-sirt1) were all synthesized by GenePharma Co. Ltd. (Shanghai, China). Breast cancer cells (1  ×  10^5^) were plated into 6-well plates and cultured in medium without antibiotics for approximately 24 h prior to transfection. The next day, cells were transiently transfected with miRNAs, siRNAs or plasmids using Lipofectamine 2000 (Invitrogen). Cells were collected 48 h post-transfection for functional analysis.

### Radiation treatment

Breast cancer cells were plated in 25 cm^2^ polystyrene flasks 24 h before radiation and then cells were exposed to irradiation with single dose of 0, 2, 4, 6 or 8 Gy using a 6 MeV electron generated by a Cs-137 irradiator (HWMD-2000, Siemens, Germany) at a dose rate of 2.4 Gy/min.

### Quantitative real-time PCR (qRT-PCR) analysis

Total RNA were isolated from cultured cells using the ISOGEN reagent (Nippon Gene, Toyama, Japan). For the detection of miR-22 and sirt1 mRNA expression, total RNA was reversely transcribed using TaqMan miRNA reverse transcription kit (Applied Biosystems Inc., Foster City, CA, USA) or the PrimeScript RT reagent kit (Takara bio, Japan), respectively. Expression levels of miR-22 and sirt1 were detected using TaqMan microRNA Assay Kit (Applied Biosystems) and Fast SYBR Green Master Mix (Applied Biosystems) on an ABI 7900HT System, respectively. miR-22 and sirt1 expressions were normalized to U6 small nuclear RNA and GAPDH using the 2^−∆∆Ct^ method. The primer sequences were presented as follows: miR-22 (forward) 5′-GGGGGATCCCTGGGGCAGGACCCT-3′, (reverse) 5′-GGGGAATTCAACGTATCATCCACCC-3′; sirt1 (forward) 5′-GCCAGAGTCCAAGTTTAGAAGA-3′, (reverse) 5′-CCATCAGTCCCAAATCCAG-3′; U6 (forward) 5′-GCTTCGGCAGCACATATACTAAAAT-3′, (reverse) 5′-CGCTTCACGAATTTGCGTGTCAT-3′; GAPDH (forward) 5′-TGGAAGGACTCATGA CCACA-3′, (reverse) 5′-TTCAGCTCAGGGATGACCTT-3′.

### Western Blot analysis

Total proteins from breast cancer cells were lysed in modified RIPA lysis buffer (Beyotime, China) with freshly added protease inhibitors cocktail (Roche Diagnostics, Basel, Switzerland) and quantified by a BCA protein assay kit (Thermo Scientific, Rockford, IL, USA). Then, 20 µg of total cellular extracts were separated by 10% SDS-PAGE and immobilized on polyvinylidene fluoride membrane (PVDF; EMD Millipore, Billerica, MA, USA). Following blocked by 5% skimmed milk (Sigma) for 2 h, the membrane was probed with primary antibodies against sirt1 and β-Actin (Abcam, Cambridge, MA, USA) overnight at 4 °C. Subsequently, the membrane was incubated with horseradish peroxidase (HRP)-conjugated goat-anti-mouse IgG (Santa Cruz biotechnology, Santa Cruz, CA, USA) for 1 h at room temperature. The protein bands were visualized using ECL detection reagent (Millipore, Billerica, MA, USA).

### Colony formation assay

Cells transfected with miR-22, miR-NC, si-sirt1, si-NC, or miR-22 + pcDNA-sirt1 were seeded into 12-well plates. An appropriate number of 2000 cells were plated into 60 mm^2^ culture dish for 12 h and then exposed to radiation at 0, 2, 4, 6 or 8 Gy. After culturing for 13 days post-irradiation, cells were fixed with 100% methanol and stained with 1% crystal violet (Sigma). Colonies containing more than 50 cells were counted manually and survival fraction were determined as below: surviving fraction  =  number of colonies counted/number of cells plated.

### γ-H2AX foci formation assay

Following transfection with miR-22, si-NC or respective control for 48 h, the cells were placed on chamber slides for overnight incubation and then treated with 6 Gy radiation. The cells were fixed for 30 min in 4% paraformaldehyde (Sigma) at 24 h post-irradiation, permeabilized in 0.1% Triton X-100 (Sigma) for 15 min, blocked for 1 h in 1% goat serum and continually incubated overnight at 4 °C with the anti-γ-H2AX primary antibody (Epitomics, Burlingame, CA, USA). The slides were then washed with PBS and incubated with fluorescein isothiocyanate conjugated secondary antibody (Santa Cruz Biotechnology) at 37 °C for 1 h. Finally, the cells were washed three times with PBS and mounted with DAPI mounting media (Invitrogen). Immunofluorescence staining was detected using a fluorescence microscope (Olympus, Shinjuku-ku, Tokyo, Japan).

### Cell viability assay

Cells were placed into 96-well plates at a concentration of 5  ×  10^4^ cells per well and cultured for 48 h. 10 μl of CCK-8 (Dojindo Molecular Technologies, Inc., Kumamoto, Japan) was then added to incubate for another 4 h at 37 °C. The optical density at 450 nm was determined with a microplate reader (Bio-Rad, Gaithersburg, MD, USA).

### Cell apoptosis assay

Approximately 5 × 10^5^ cells were harvested 48 h post-transfection, washed three times with PBS, and resuspended in 100 μl binding buffer. The cells were then incubated with 5 μl fluorescein FITC-conjugated Annexin V for 10 min and 5 μl propidium iodide (PI; KeyGen, Nanjing, China) for 15 min in the dark. The apoptotic cells were analyzed by a BD FACSCanto flow cytometer (BD Biosciences, San Jose, CA, USA).

### Luciferase reporter assay

The sequences of 3′-UTR of wild type and mutant sirt1 mRNA containing the putative miR-22 binding sites were chemically synthesized from GeneChem and cloned into downstream of the luciferase gene in the pGL3 vectors (Promega, Madison, WI, USA) to generate the vectors pGL3-sirt1-3′UTR-WT and pGL3-sirt1-3′UTR-MUT. For luciferase reporter assay, cells were plated in 6-well plates and cotransfected with 2  μg of luciferase constructs and 10 pmol of miR-22 or miR-NC using Lipofectamine 2000 (Invitrogen). At 24 h post-transfection, cells were harvested for detection of firefly luciferase activity using the Dual Luciferase Reporter Assay System (Promega).

### Statistical analysis

All data were shown as mean ± SD. All statistical analyses were performed with Student’s *t* test and one-way ANOVA using SPSS 12.0 computer software (SPSS Inc., Chicago, IL, USA). Differences were considered statistically significant at *P* values  < 0.05.

## Results

### miR-22 was downregulated and sirt1 was upregulated in breast cancer cells

**Fig. 1:**
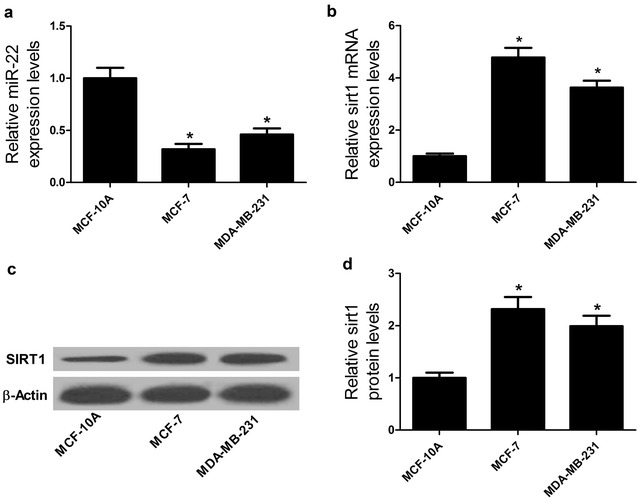
miR-22 was downregulated and sirt1 was upregulated in breast cancer cells. qRT-PCR analyses were performed to detect the expression levels of miR-22 (**a**) and sirt1 mRNA (**b**) in breast cancer cell lines (MCF-7 and MDA-MB-231) and normal breast epithelial cell line MCF-10A. **c**, **d** Western Blot was carried out to determine the protein level of sirt1 in MCF-7, MDA-MB-231 and MCF-10A. *P < 0.05

To explore the role of miR-22 and sirt1 in the development of breast cancer, we analyzed the expressions of miR-22 and sirt1 at mRNA and protein levels in breast cancer cells by qRT-PCR and Western Blot. As illustrated in Fig. 1a, b, qRT-PCR results demonstrated that miR-22 expression was dramatically lower and sirt1 mRNA was markedly higher in breast cancer cell lines MCF-7 and MDA-MB-231 than that in normal breast epithelial cell line MCF-10A. Meanwhile, the protein level of sirt1 was significantly elevated in both MCF-7 and MDA-MB-231 cells compared with that in MCF-10A cells (Fig. 1c, d), as demonstrated by Western Blot. Therefore, we supposed that miR-22 and sirt1 may be associated with the development of breast cancer.

### miR-22 overexpression suppressed tumorigenesis and improved radiosensitivity of breast cancer cells

**Fig. 2:**
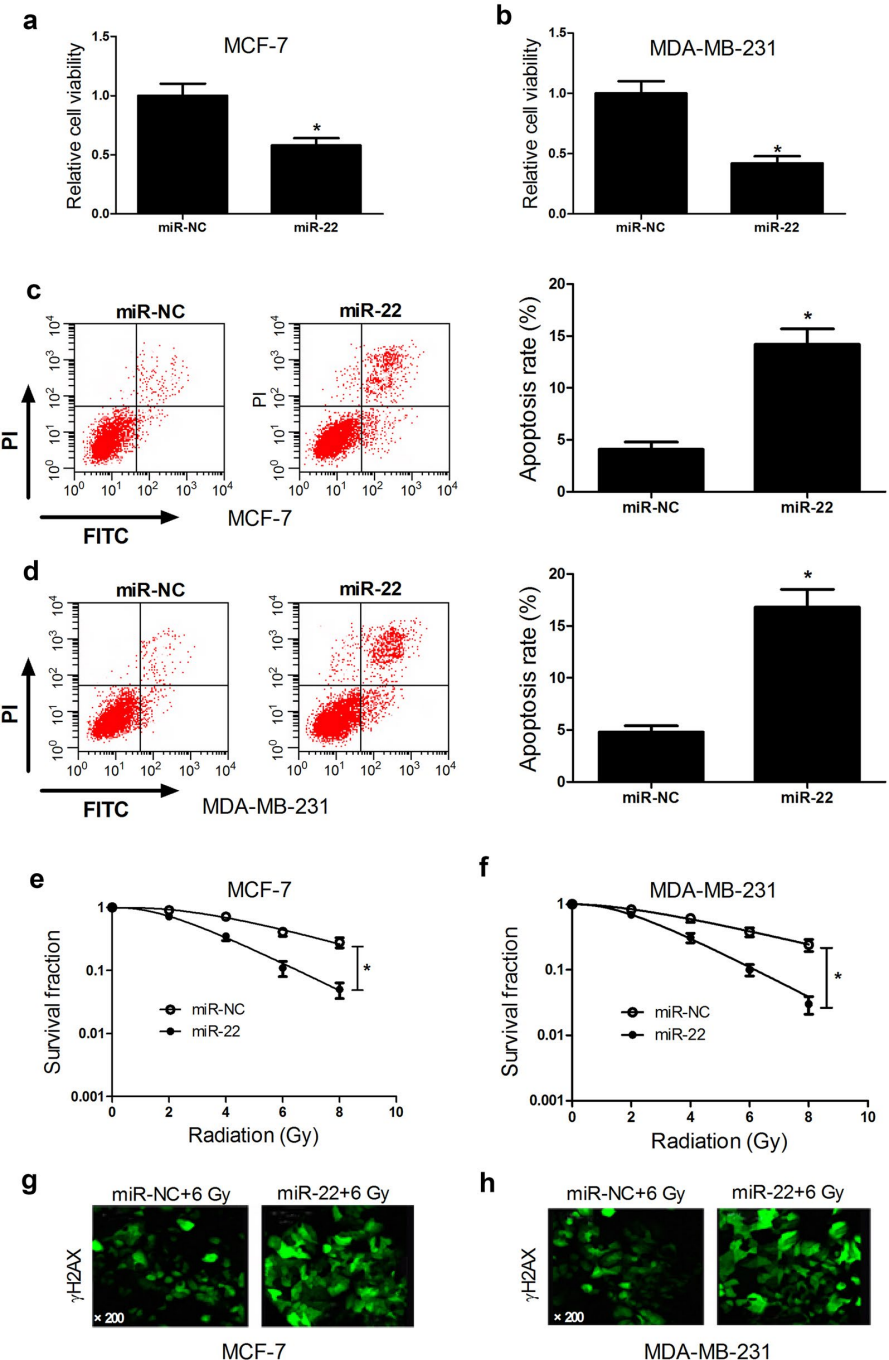
Effect of miR-22 overexpression on tumorigenesis and radiosensitivity of breast cancer cells. MCF-7 and MDA-MB-231 cells were transfected with miR-22 or miR-NC and cultured for 48 h. Cell viability in transfected MCF-7 (**a**) and MDA-MB-231 (**b**) cells were examined by CCK-8 assay. Apoptosis of transfected MCF-7 (**c**) and MDA-MB-231 (**d**) cells was assessed by flow cytometry analysis. Colony formation assay was performed to detect survival fraction in transfected MCF-7 (**e**) and MDA-MB-231 (**f**) cells with indicated doses of irradiation (0, 2, 4, 6, or 8 Gy). γ-H2AX foci formation assay was carried out to detect the number of γ-H2AX foci in transfected MCF-7 (**g**) and MDA-MB-231 (**h**) cells with 6 Gy radiation. *P < 0.05

To further identify the biological role of miR-22 in breast cancer cells, we performed gain-of-function experiments in MCF-7 and MDA-MB-231 cells by transfecting with miR-22 mimic. CCK-8 assay and flow cytometry analysis were performed to examine the effect of ectopic expression of miR-22 on tumorigenesis of breast cancer cells. CCK-8 assay results revealed that miR-22 overexpression led to a dramatic decrease of cell viability in MCF-7 (Fig. 2a) and MDA-MB-231 (Fig. 2b) cells compared with miR-NC group. Flow cytometry analysis showed that enforced expression of miR-22 significantly increased apoptosis rates of MCF-7 (Fig. 2c) and MDA-MB-231 (Fig. 2d) cells compared with that of controls. Colony formation assay was used to evaluate the effect of miR-22 overexpression on radiosensitivity of breast cancer cells. The results suggested that exogenous expression of miR-22 obviously reduced the survival fraction of MCF-7 (Fig. 2e) and MDA-MB-231 (Fig. 2f) cells with respect to miR-NC-transfected cells, suggesting that miR-22 overexpression increased radiosensitivity of breast cancer cells. The γ-H2AX foci is a sensitive marker of DNA double-strand break (DSB) induced by radiation [24]. Therefore, to explore the effect of miR-22 overexpression on repair ability of DNA damage, γ-H2AX foci formation assay following radiation was employed. As shown in Fig. 2g, h, the number of γ-H2AX foci was dramatically increased in miR-22-transfected MCF-7 and MDA-MB-231 cells after 6 Gy irradiation in comparison with miR-NC group, suggesting that miR-22 overexpression suppressed irradiation-induced DNA damage repair. Collectively, these results indicated that miR-22 overexpression suppressed tumorigenesis by inhibiting proliferation and promoting apoptosis and improved radiosensitivity of breast cancer cells by restraining DNA damage repair.

### Sirt1 knockdown inhibited tumorigenesis and enhanced radiosensitivity of breast cancer cells

**Fig. 3:**
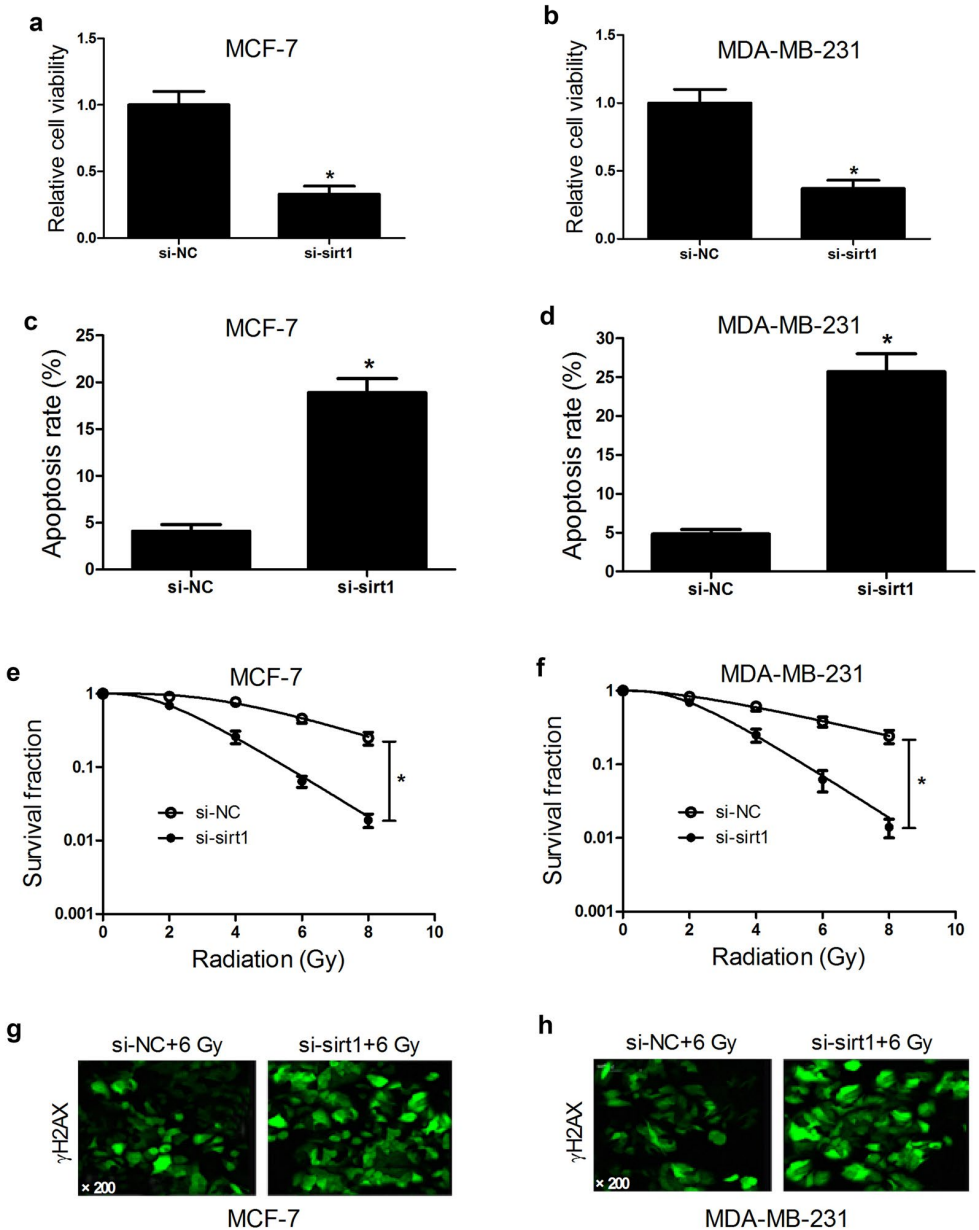
Effect of sirt1 knockdown on tumorigenesis and radiosensitivity of breast cancer cells. MCF-7 and MDA-MB-231 cells were transfected with si-sirt1 or si-NC and cultured for 48 h. Cell viability was detected by CCK-8 assay in transfected MCF-7 (**a**) and MDA-MB-231 (**b**) cells. Apoptosis was evaluated by flow cytometry analysis in transfected MCF-7 (**c**) and MDA-MB-231 (**d**) cells. Colony formation assay was used to detect survival fraction in transfected MCF-7 (**e**) and MDA-MB-231 (**f**) cells exposed to 0, 2, 4, 6, or 8 Gy of irradiation. The expression of γ-H2AX in transfected MCF-7 (**g**) and MDA-MB-231 (**h**) cells with 6 Gy radiation was detected by γ-H2AX foci formation assay. *P < 0.05

To evaluate the role of sirt in tumorigenesis and radiosensitivity of breast cancer cells, siRNA-mediated sirt1 knockdown was carried out in MCF-7 and MDA-MB-231 cells. As demonstrated by CCK-8 assay, cell viability was significantly reduced in si-sirt1-transfected MCF-7 (Fig. 3a) and MDA-MB-231 (Fig. 3b) cells compared to control group. Meanwhile, sirt1 knockdown led to a significant increase of apoptosis rates in MCF-7 (Fig. 3c) and MDA-MB-231 (Fig. 3d) cells in contrast to si-NC group. Moreover, colony formation assay showed that survival fractions of si-sirt1-transfected MCF-7 (Fig. 3e) and MDA-MB-231 (Fig. 3f) cells were dramatically suppressed after radiation compared with si-NC group. Furthermore, γ-H2AX expression in si-sirt1-transfected MCF-7 (Fig. 3g) and MDA-MB-231 (Fig. 3h) cells was also improved following irradiation compared with si-NC group. Taken together, we concluded that sirt1 knockdown repressed tumorigenesis by blocking proliferation, inducing apoptosis and increased radiosensitivity of breast cancer cells by restraining DNA damage repair.

### Sirt1 was a target of miR-22 in breast cancer cells

**Fig. 4:**
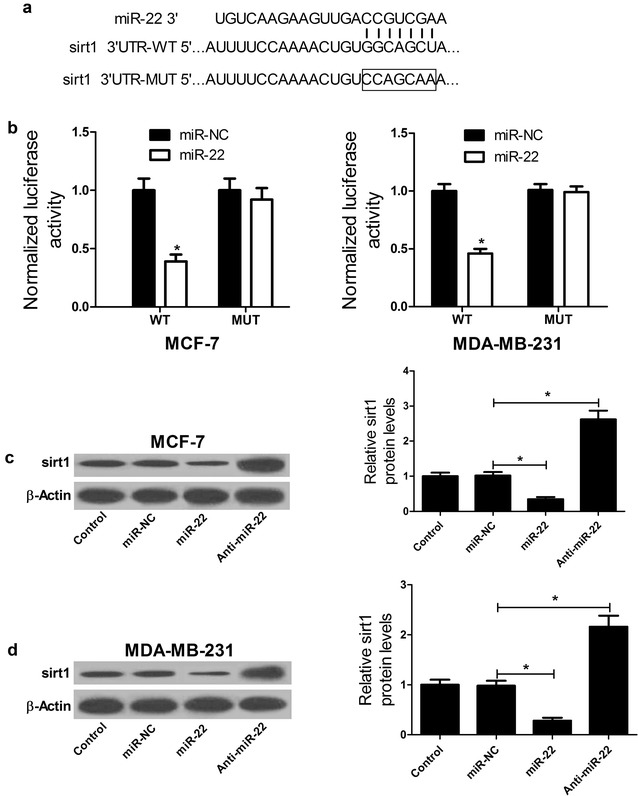
Sirt1 was a target of miR-22 in breast cancer cells. **a** The predicted binding sites of sirt1 3′UTR in miR-22 sequence and the mutations in the binding region are shown. **b** The luciferase activity was determined by luciferase reporter assay after MCF-7 and MDA-MB-231 cells were cotransfected with luciferase constructs and miR-22 or miR-NC. qRT-PCR was employed to assess the expressions of sirt1 in MCF-7 (**c**) and MDA-MB-231 (**d**) cells transfected with miR-NC, miR-22 or anti-miR-22. *P < 0.05

We further investigated the underlying mechanism by which miR-22 exerted its biological functions in breast cancer cells. Previous studies have demonstrated that sirt1 was a target of miR-22 [21, 23]. To confirm whether miR-22 could directly target sirt1 in breast cancer cells, we constructed luciferase reporter vectors containing the wild type or mutant miR-22 binding sites in the 3′UTR of sirt1 (Fig. 4a). Luciferase reporter assay results showed that transfection of miR-22 led to a significant decrease of luciferase reporter activity of sirt1-3′UTR-WT in MCF-7 and MDA-MB-231 cells, but not affect the luciferase expression of sirt1-3′UTR-MUT (Fig. 4b). To further explore whether miR-22 could negatively regulate the expression of sirt1, qRT-PCR was performed to examine the expression of sirt1 expression in MCF-7 and MDA-MB-231 cells transfected with miR-22, anti-miR-22 or miR-NC. As expected, miR-22 overexpression triggered a marked reduction of sirt1 expression in both MCF-7 (Fig. 4c) and MDA-MB-231 (Fig. 4d) cells compared with miR-NC group. Reversely, inhibition of miR-22 resulted in an obvious improvement of sirt1 expression. These data demonstrated that miR-22 directly targeted 3′UTR of sirt1 and negatively regulated its expression in breast cancer cells.

### miR-22 suppressed tumorigenesis and improved radiosensitivity of breast cancer cells by targeting sirt1

**Fig. 5:**
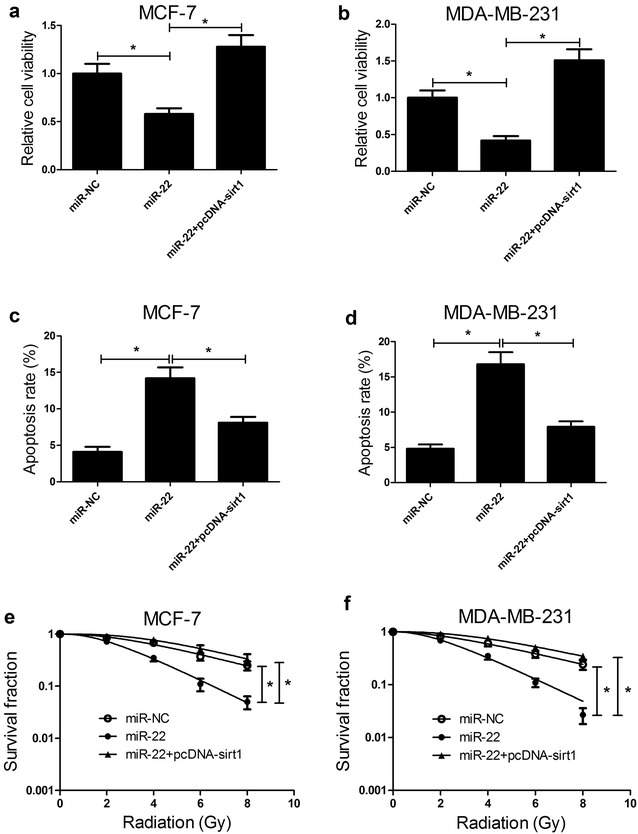
miR-22 suppressed tumorigenesis and improved radiosensitivity of breast cancer cells by targeting sirt1. MCF-7 and MDA-MB-231 cells were transfected with miR-22, miR-NC or combined miR-22 and pcDNA-sirt1 and further experiment analyses were carried out 48 h post-transfection. Cell viability of transfected MCF-7 (**a**) and MDA-MB-231 (**b**) cells were assessed by CCK-8 assay. Flow cytometry analysis was performed to determine apoptosis in transfected MCF-7 (**c**) and MDA-MB-231 (**d**) cells. Colony formation assay was carried out to calculate survival fractions in transfected MCF-7 (**e**) and MDA-MB-231 (**f**) cells after treatment with different single doses of irradiation (0, 2, 4, 6, or 8 Gy). *P < 0.05

In order to verify whether miR-22 exerted its biological roles by regulating sirt1, MCF-7 and MDA-MB-231 cells were transfected with miR-22, miR-NC or miR-22 + pcDNA-sirt1. As demonstrated by CCK-8 assay, miR-22 overexpression significantly suppressed cell viability in MCF-7 (Fig. 5a) and MDA-MB-231 (Fig. 5b) cells while transfection of pcDNA-sirt1 markedly abolished this effect. The results from flow cytometry analysis showed that miR-22-transfected MCF-7 (Fig. 5c) and MDA-MB-231 (Fig. 5d) cells exerted higher apoptotic rates than miR-NC group. In contrast, ectopic expression of sirt1 dramatically attenuated the promotion effect on apoptosis triggered by miR-22 in MCF-7 and MDA-MB-231 cells. Colony formation assay demonstrated that the survival fraction of MCF-7 (Fig. 5e) and MDA-MB-231 (Fig. 5f) cells transfected with miR-22 was conspicuously decreased compared with miR-NC group, which was reversed by sirt1 overexpression. Taken together, these results indicated that sirt1 overexpression reversed miR-22-mediated suppression on tumorigenesis and enhancement on radiosensitivity of breast cancer cells.

## Discussion

In the present study, we demonstrated the downregulation of miR-22 and upregulation of sirt1 in breast cancer cells. Ectopic expression of miR-22 and sirt1 knockdown both suppressed cell viability, promoted apoptosis and increased radiosensitivity of breast cancer cells. Notably, sirt1 was also identified as a direct target of miR-22 in breast cancer cells. Moreover, ectopic expression of sirt1 significantly overturned the suppressive effect on tumorigenesis and promotion effect on radiosensitivity of breast cancer cells mediated by miR-22 overexpression, indicating that miR-22 played a tumor suppressive role by targeting sirt1 in breast cancer cells.

A growing body of evidence has indicated that dysregulation of miR-22 is implicated in the regulation of various tumor progressions. Forced expression of miR-22 repressed proliferation, colony formation, migration and invasion of gastric cancer cells by targeting CD151 [25]. miR-22 suppressed osteosarcoma cell proliferation and migration by targeting HMGB1 and inhibiting HMGB1-mediated autophagy [26]. miR-22 downregulation participated in tumorigenicity and progression of hepatocellular carcinoma cells through upregulating histone deacetylase 4 (HDAC4) expression [27]. A previous study has reported that miR-22 functioned as a tumor suppressor in breast cancer cells and may be a promising prognostic biomarker in breast cancer [28]. The present study used MCF-7 cells as an in vitro model for ER-positive breast cancer, and MDA-MB-231 cells as an in vitro model of ER-negative breast cancer to avoid the deviation. In accordance with the previous study, our study demonstrated that miR-22 expression was downregulated and restoration expression of miR-22 suppressed tumorigenesis of MCF-7 and MDA-MB-231 cells by inhibiting cell viability and inducing apoptosis. More notably, we found that miR-22 overexpression enhanced radiosensitivity of breast cancer cells by restraining DNA damage repair. Consistently, a previous study showed that progesterone-treatment and irradiation triggered downregulation of miR-22 expression, causing an increase in proportions of radiation-resistant tumor-initiating of cancer stem cells in breast cancer [29].

Sirt1, a member of the mammalian sirtuin family, plays a pivotal role in the modulation of various metabolic pathways [30]. It is well established that sirt1 serves as a crucial regulator in diverse biological processes, including apoptosis, cell growth, DNA damage and tumor development in mammalian [31]. Moreover, it has been shown that downregulation of sirt1 expression in vitro by antisense oligonucleotides or in vivo by siRNA enhances radiation sensitization in cancerous cells, as well as radiation-induced apoptosis [32, 33]. Accumulating evidence reveals that sirt1 promotes the tumorigenesis of various cancers, such as breast cancer, gastric cancer [34, 35]. Our study confirmed the increased expression of sirt1 in breast cancer cells. Results from loss-of-function strategy demonstrated that sirt1 played an oncogenic role in breast cancer cells. Sirt1 knockdown dramatically improved radiosensitivity of breast cancers by suppressing DNA damage repair. Many studies have indicated that various miRNAs including miR-34a [36], miR-22 [21], and miR-494 [37] can directly target sirt1 and regulate sirt1 expression and function. As expected, sirt1 was identified as a direct target of miR-22 and miR-22 negatively regulated sirt1 expression. Functional analysis further demonstrated that ectopic expression of sirt1 significantly reversed miR-22-mediated suppression on tumorigenesis and enhancement on radiosensitivity of breast cancer cells, suggesting that miR-22 inhibited tumorigenesis and increased radiosensitivity of breast cancer cells by targeting sirt1.

## Conclusions

In conclusion, our study demonstrated that miR-22 expression was downregulated and sirt1 was upregulated in breast cancer cells. Further studies revealed that exogenous expression of miR-22 suppressed tumorigenesis and improved radiosensitivity of breast cancer cells by targeting sirt1. Therefore, miR-22 may be a promising therapeutic target for the treatment of breast cancer.

## Abbreviations

miRNAs: microRNAs
Sirt1: silent information regulator 1
EGF: epidermal growth factor
qRT-PCR: quantitative real-time PCR
HRP: horseradish peroxidase
DSB: double-strand break
HDAC4: histone deacetylase 4
